# The effect of CpG-ODN on antigen presenting cells of the foal

**DOI:** 10.1186/1476-8518-5-1

**Published:** 2007-01-25

**Authors:** M Julia BF Flaminio, Alexandre S Borges, Daryl V Nydam, David W Horohov, Rolf Hecker, Mary Beth Matychak

**Affiliations:** 1 Department of Clinical Sciences, College of Veterinary Medicine, Cornell University, Ithaca, NY, USA; 2 Departamento de Clinica Veterinaria, Faculdade de Medicina Veterinaria e Zootecnia, Universidade Estadual Paulista 'Julio de Mesquita Filho', UNESP-Campus de Botucatu, SP, Brazil; 3 Department of Population Medicine and Diagnostics Sciences, College of Veterinary Medicine, Cornell University, Ithaca, NY, USA; 4 Department of Veterinary Science, Maxwell H. Gluck Equine Research Center, University of Kentucky, Lexington, KY, USA; 5 Qiagen GmbH, Hilden, Germany; current address Tübingen, Germany

## Abstract

**Background:**

Cytosine-phosphate-guanosine oligodeoxynucleotide (CpG-ODN) has been used successfully to induce immune responses against viral and intracellular organisms in mammals. The main objective of this study was to test the effect of CpG-ODN on antigen presenting cells of young foals.

**Methods:**

Peripheral blood monocytes of foals (n = 7) were isolated in the first day of life and monthly thereafter up to 3 months of life. Adult horse (n = 7) monocytes were isolated and tested once for comparison. Isolated monocytes were stimulated with IL-4 and GM-CSF (to obtain dendritic cells, DC) or not stimulated (to obtain macrophages). Macrophages and DCs were stimulated for 14–16 hours with either CpG-ODN, LPS or not stimulated. The stimulated and non-stimulated cells were tested for cell surface markers (CD86 and MHC class II) using flow cytometry, mRNA expression of cytokines (IL-12, IFNα, IL-10) and TLR-9 using real time quantitative RT-PCR, and for the activation of the transcription factor NF-κB p65 using a chemiluminescence assay.

**Results:**

The median fluorescence of the MHC class II molecule in non-stimulated foal macrophages and DCs at birth were 12.5 times and 11.2 times inferior, respectively, than adult horse cells (p = 0.009). That difference subsided at 3 months of life (p = 0.3). The expression of the CD86 co-stimulatory molecule was comparable in adult horse and foal macrophages and DCs, independent of treatment. CpG-ODN stimulation induced IL-12p40 (53 times) and IFNα (23 times) mRNA expression in CpG-ODN-treated adult horse DCs (p = 0.078), but not macrophages, in comparison to non-stimulated cells. In contrast, foal APCs did not respond to CpG-ODN stimulation with increased cytokine mRNA expression up to 3 months of age. TLR-9 mRNA expression and NF-kB activation (NF-kB p65) in foal DCs and macrophages were comparable (p > 0.05) to adult horse cells.

**Conclusion:**

CpG-ODN treatment did not induce specific maturation and cytokine expression in foal macrophages and DCs. Nevertheless, adult horse DCs, but not macrophages, increased their expression of IL-12 and IFNα cytokines upon CpG-ODN stimulation. Importantly, foals presented an age-dependent limitation in the expression of MHC class II in macrophages and DCs, independent of treatment.

## Background

The susceptibility of the naïve foal to infection in the neonatal period is greatly dependent on the adequacy of transfer and absorption of maternally-derived antibodies through the colostrum. Passively-transferred humoral immune protection, though, is limited and short-lived. When maternal antibodies are reduced to low levels, the foal must rely on its immune system to resist infections. In addition, protection against intracellular pathogens may require cellular immunity. Therefore, early maturation of the foal's immune system would likely increase resistance to infectious disease.

Bacterial DNA has a potent immunostimulatory activity explained by the presence of frequent unmethylated cytosine-phosphate-guanosine (CpG) motifs [1,2]. Synthetic CpG-oligodeoxynucleotides (CpG-ODN) have shown potent immunostimulatory activity in adult and in neonatal vertebrates likely because they mimic bacterial DNA [3]. *In vivo*, CpG-ODNs have been shown to induce strong Type 1 immune responses, with subsequent activation of cellular (cytotoxic T lymphocytes, CTLs) and humoral (Th1 immunoglobulin isotypes) components [4]. Therefore, CpG-ODNs have been extensively studied for their application as adjuvants in vaccines in domestic species, including bovine, ovine and swine, revealing increase in vaccine efficacy and protection [5-11]. In the horse, CpG-ODN 2007 formulated in 30% Emulsigen added to a commercial killed-virus vaccine against equine influenza virus enhanced the antibody responses in comparison to the vaccine alone [12].

Toll-like receptors (TLRs) are essential for the recognition of highly conserved structural motifs (pathogen-associated molecular patterns or PAMPS) only expressed by microbial pathogens. The combination of different TLRs provides detection of a wide spectrum of microbial molecules. For instance, TLR-4 specifically recognizes lipopolysaccharide (LPS) derived from gram-negative bacteria, whereas bacterial DNA (unmethylated CpG motif) is recognized by TLR-9 [13]. TLRs are predominantly expressed on antigen-presenting cells [macrophages, dendritic cells (DCs) and, to some extent, B cells], which are abundantly present in immune tissues (spleen, lymph nodes, peripheral blood leukocytes), as well as tissues that are directly exposed to microorganisms (lungs, gastrointestinal tract, skin). The nuclear-factor kB (NF-kB) is a transcription factor activated upon recruitment of the adaptor MyD88 and TLR 4 or TLR9 engagement with PAMPs [14]. Antigen presenting cells (APCs) play a major role in the initiation and instruction of antigen-specific immune response, and are the link between innate and adaptive immunity: they recognize, process and present antigen to T cells. Many studies have indicated that DCs, but not macrophages, are critical for the induction of primary immune responses, i.e. a first time T cell encounter with processed antigen [15]. Dendritic cells ability to process and present antigen depends on their stage of maturation, and circulating precursor DCs enter tissues as immature DCs. After antigen capture, they migrate to secondary lymphoid organs where they become mature DCs. Immature DCs exhibit active phagocytosis but lack sufficient cell surface MHC class II and co-stimulatory molecules (CD83, CD86) for efficient antigen presentation to T lymphocytes [16]. In contrast, mature DCs demonstrate decreased capacity of phagocytosis and antigen processing, and increased expression of MHC class II and co-stimulatory molecule on the cell surface. CpG-ODNs have been shown to induce maturation of DCs by increasing cell surface expression of MHC class II, CD40, and CD86/80 molecules [17]. In combination with antigens, CpG-ODNs enhance antigen processing and presentation by DCs and the expression of Type I cytokines (i.e. type I interferon IFNα and IL-12) [18]. In the horse, Wattrang et al. (2005) demonstrated that phosphodiester ODN containing unmethylated CpG-ODN motif induced type I interferon production in peripheral blood mononuclear cells [19]. Activation of human monocytes through Toll-like receptor has been shown to induce their differentiation into either macrophages or DCs, and the presence of GM-CSF is synergistic for the expression of MHC class II, CD86, CD40 and CD83 molecules, mixed lymphocyte reaction and the secretion of Th1 cytokines by T cells [20].

In contrast to adults, human neonates have demonstrated impaired response to multiple PAMPS, which may significantly contribute to immature neonatal immunity [21,22]. Nevertheless, CpG-ODN has been shown to induce *in vitro *IFNα cytokine production and reduce *in vivo *viral shedding in newborn lambs [23]. To date, limited information is available about the competence of foal cells to detect pathogens and trigger an immune response against them. A similar dependency in APC competency could exist in the foal in regards to resistance to viral and intracellular bacterial infections, for instance *Rhodococcus equi*, which causes pyogranulomatous pneumonia exclusively in young foals [24,25].

The *ex vivo *system used in this investigation allowed a longitudinal study of the immune cells of the foal. We investigated the effect of a CpG-ODN on monocyte-derived macrophages and DCs from adult horses and foals from birth to 3 months of life. We evaluated the effect of CpG-ODN in the maturation process of dendritic cells of foals and compared to those of adult horses by measuring cell surface molecule expression, cytokine profile, and signaling pathway activation.

## Methods

### Foals, adult horses and blood samples

This study was conducted following a protocol approved by Cornell University Center for Animal Resources and Education and the guidelines from the Institutional Animal Care and Use Committees. Eight pregnant mares of various breeds (1 Bavarian, 1 Westfalen, 1 Selle Fraincaise, 1 Thoroughbred, 2 Oldenburg, 2 Pony mares) belonging to the Cornell University Equine Park were monitored for this study. Those mares had access to pasture and barn, and they were fed grass hay and grain according to their management schedule. They were vaccinated approximately 30 days before foaling with Encevac-T^® ^(Intervet, DeSoto, KS). All the foalings were observed, and the adequate absorption of colostral immunoglobulin G (IgG) by the foals was assessed using the SNAP^® ^Test (Idexx, Westbrook, MN) by 18 hours of birth. Daily physical examination in the first week of life, and monthly complete blood cell count were performed to evaluate natural inflammatory/infectious conditions in the foals.

Sixty milliliter peripheral blood samples were collected from the 8 foals via jugular venipuncture using heparinized vacutainer tubes within 5 days of life, and monthly up to 3 months of life. One of the foals was euthanized due to septic synovitis and was removed from the study. An equivalent amount of blood was collected once from 7 different adult horses (5 Thoroughbred and 2 ponies). All the samples were processed as below immediately after collection.

### Monocyte-derived macrophages and dendritic cells

Monocytes were purified from peripheral blood using a modified technique described by Hammond et al. [26]. Briefly, mononuclear cells were isolated using Ficoll-Paque (Amershan Biosciences, Piscataway, NJ) density centrifugation, and incubated in DMEM-F12 medium (Gibco-Invitrogen Corporation, Grand Island, NY) plus 5% bovine growth serum (Hyclone, Logan UT), antibiotics and antimycotics (Gibco-Invitrogen Corporation, Grand Island, NY) for 4 h at 5% CO_2_, 37°C. All those reagents were certified for the presence of lipopolysaccharide. The loosely adherent and non-adherent cells were removed by gentle wash with 37°C phosphate buffered solution (PBS). For the generation of DCs, recombinant equine IL-4 (rEqIL-4, 10 ng/ml) and recombinant human granulocyte-monocyte colony stimulating factor (rHuGM-CSF, 1000 units/ml, R&D Systems, Minneapolis, MN) were added to the culture medium as the following:

*Dendritic cell baseline control: *for the generation of DCs, monocytes were cultured in the presence of rEqIL-4 and rHuGM-CSF for 5 days.

*To test the effect of CpG-ODN or LPS on dendritic cells: *monocytes were cultured in the presence of rEqIL-4 (10 ng/ml) and rHuGM-CSF (1,000 units/ml) for 5 days, followed by the addition of CpG-ODN 1235 (10 μg/ml, Qiagen, Hilden, Germany) or LPS (Sigma Diagnostics, Inc., St. Lois, MO) to the medium for 14–16 hours.

*Macrophage baseline control: *monocytes were cultured with no extra additives for 5 days.

*To test the effect of CpG-ODN or LPS on macrophages: *monocytes were cultured with no extra additives for 5 days, followed by the addition of CpG-ODN 2135 (10 μg/ml) or LPS (12.5 μg/ml) to the medium for 14–16 hours.

Cell viability (> 90%) and morphology (formation of dendrites) were tested by 0.2% Trypan blue (Gibco BRL, Grand Island, NY) exclusion and contrast phase microscopy, respectively. One portion of the cultured cells was tested for cell surface molecule expression using flow cytometry. The adhered cells were detached from the wells using 5 mM EDTA in medium for 5–10 minutes at 37°C, and washed with fresh PBS. The plates were evaluated afterward to ensure all cells were removed for analysis. In general, macrophages presented moderate adherence to the plates, whereas dendritic cells were loose or loosely attached. The other portion was snap frozen in liquid nitrogen and stored at minus 80°C for: a) RNA extraction, and subsequent measurement of gene expression using real-time RT-PCR; or b) measurement of NF-κB activation using a chemiluminescence assay.

### Unmethylated cytosine-phosphate-guanosine oligodeoxynucleotides (CpG-ODN) motifs

In this study, we used the synthetic CpG-ODN 2135 (TCGTCGTTTGTCGTTTTGTCGTT) (Merial, USA), which has been shown to induce equine peripheral blood mononuclear cell proliferation *in vitro *[27]. To confirm the recognition of this CpG-ODN motif by horse peripheral blood leukocytes and collect preliminary data about the response in foals, 2-day-old foal (n = 5) and adult horse (n = 5) isolated peripheral blood mononuclear cells, and a 5-day-old foal isolated mesenteric lymph node mononuclear cells (n = 1) were cultured in the presence or absence of 5 μg/ml or 10 μg/ml CpG-ODN 2135, 12.5 μg/ml LPS or non-stimulated. Approximately 4 × 10^5 ^cells/well were cultured in a 96-well plate and medium described above. The cells were incubated for 3 days at 37°C in 5% CO_2_, and pulsed with 0.8 μCi [^3^H]-thymidine per well for the last 8 hours of incubation. Well contents were harvested onto glass fiber filters and [^3^H]-thymidine incorporation was measured using a liquid scintillation beta counter. The stimulation index was calculated dividing the average counts per minute from stimulated cells by the average counts per minute from non-stimulated cells.

### Flow cytometric analysis of cell surface markers

Cell surface markers of monocyte-derived macrophages and DCs were evaluated by flow cytometry after 5 days of culture (Day 5) and after overnight stimulation with CpG-ODN or LPS (Day 6). The assay was performed according to Flaminio et al. [28], and monoclonal antibodies used are described in Table 1[29-31]. Leukocyte subpopulations were displayed in a dot plot and gated according to size based on forward light scatter (FSC), and according to granularity based on 90 degree side light scatter (SSC). The cell population of interest was gated away from small and dead cells, including events greater than 400 FSC and 200 SSC. Both percentage positive cells and mean fluorescence expression were measured.

**Table 1:** Monoclonal antibodies used to test the expression of cell surface markers of monocyte-derived macrophages and dendritic cells stimulated or not with CpG-ODN or LPS

**MARKER**	**ANTIBODY**	**CLONE**	**SUPPLIER**	**VALIDATION**
CD172a	mouse anti-bovine CD172a	DH59B	VMRD, Pullman, WA	Kydd et al., 1994
CD86	mouse anti-human CD86	2331(FUN-1)	Becton and Dickinson, San Diego, CA	Hammond et al., 1999
MHC I	mouse anti-horse MHC I	CZ3	D. Antczak's laboratory, Cornell University	Lunn et al., 1998
MHC II	mouse anti-horse MHC II	CZ11	D. Antczak's laboratory, Cornell University	Lunn et al., 1998
CD14	mouse anti-human CD14	big10	Biometec, Germany	Steinbach et al., 1998
Negative	mouse anti-canine parvovirus	--	C.Parrish's laboratory, Cornell University	Parrish et al., 1982

### Real-time RT-PCR reactions for cytokine mRNA expression

Quantitative analysis of cytokine mRNA expression was performed as described in Flaminio et al. [32]. Isolation of total RNA from monocyte-derived macrophages and DCs was performed using RNeasy^® ^Mini Kit (Qiagen, Valencia, CA), and quality of RNA was tested by 260/280 nm. The RNA product was treated with DNAse to eliminate possible genomic DNA from the samples, and the lack of amplification of genes in samples without the addition of reverse transcriptase confirmed the purity of RNA. A same amount (0.01 μg in 1 μL) of RNA from each sample was used to test for the expression of cytokines. The cytokine (IL-10, IL-12p35, IL-12p40 and IFNα) and Toll-like receptor 9 (TLR9) gene expression in stimulated and non-stimulated cells was measured in triplicate using Taqman^® ^one-step RT-PCR master mix reagents, specific primers and probes designed using published equine sequences (Table 2), and the ABI Prism^® ^7700 Sequence Detection System (AB Biosystems, Foster City, CA). In a small subset of adult horse cells (n = 3), the expression of TNFα mRNA was tested at 14–16 hours of culture. Analysis of data was performed by normalizing the target gene amplification value (Target C_T_) with its corresponding endogenous control (βactin, Reference C_T_). The quantity of the target gene in each sample was calculated relatively to the calibrator sample (fold difference over Day 5 non-stimulated cells).

**Table 2:** Primer and probe sequences used to measure mRNA expression in monocyte-derived macrophages and dendritic cells

**CYTOKINE**	**PRIMER AND PROBE SEQUENCES**	**GenBank accession #**
**IL-12p35**	5'-TCA AGC TCT GCA TCC TTC TTC AT-3'	Y11130
	5'-CAG ATA GCC CAT CAT CCT GTT G-3'	
	5'-FAM-CCT TCA GAA TCC GCG CAG TGA CCA-TAMRA-3'	
		
**IL-12p40**	5'-CAC CTG CAA TAC CCC TGA AGA-3'	Y11129
	5'-TGC CAG AGC CTA AGA CCT CAT T-3'	
	5'-FAM-CAT CAC CTG GAC CTC GGC CCA-TAMRA-3'	
		
**IFNα**	5'-AGG TGT TTG ACG GCA ACC A-3'	M14540
	5'-ACG AGC CGT CTG TGC TGA A-3'	
	5'-FAM-AGC CTC AAG CCA TCT CCG CGG T-TAMRA-3'	
		
**IL-10**	5'-GAC ATC AAG GAG CAC GTG AAC TC-3'	U38200
	5'-CAG GGC AGA AAT CGA TGA CA-3'	
	5'-FAM-AGC CTC ACT CGG AGG GTC TTC AGC TT-TAMRA-3'	
		
**TNFα**	5'-GAT GAC TTG CTC TGA TGC TAA TCC-3'	M64087
	5'-TCT GGG CCA GAG GGT TGA T-3'	
	5'-FAM-TCT CCC CAG CAG TTA CCG AAT GCC TT-TAMRA-3'	
		
**TLR9**	5'-AAC TGG CTG TTC CTG AAG TCT GTG-3'	DQ157779
	5'-TCA ACC TCA AGT GGA ACT GCC C-3'	
	5'-FAM-AGA GAA CTG TCC TTC AAC ACC AGG-TAMRA-3'	
		
**β-actin**	5'-TCA CGG AGC GTG GCT ACA-3'	AF035774
	5'-CCT TGA TGT CAC GCA CGA TTT-3'	
	5'-FAM-CAC CAC CAC GGC CGA-TAMRA-3'	

To determine the time-point for cell harvesting that corresponded to the approximate peak of cytokine expression in CpG-ODN stimulated cells, samples from 3 adult horses were tested at different time points for cytokine mRNA expression. Results indicated that the peak of IL-12p40 expression was at observed between 12 and 24 hours of stimulation (data not shown).

### Toll-like receptor 9 (TLR9)

Consensus sequence was obtained by aligning the human, bovine, ovine, canine, feline and murine TLR9 gene sequences using the gene alignment NTI software. Primers for the consensus sequence were designed and used for PCR amplification of horse cDNA obtained from purified peripheral blood leukocyte RNA. Gel electrophoresis of the PCR product using low melting point gel agar revealed a single band of expected size. The PCR product was purified using QIAquick PCR purification kit (Qiagen, Valencia, CA). The PCR product was ligated into the pDrive cloning vector, followed by transformation of Quiagen EZ chemically competent cells (Qiagen, Valencia, CA). Selected colonies were grown overnight and plasmid DNA was isolated with the QIAprep Spin Miniprep Kit (Qiagen, Valencia, CA). Inserts were confirmed with restriction digest and/or PCR. Desired clones were sequenced with universal primers at Cornell University Sequencing Center. Primers and probes were designed for the quantitative RT-PCR using the equine sequence and the PrimerExpress software (ABIPrism). The equine TLR9 partial sequence was submitted to GenBank under accession number DQ157779.

### Nuclear-factor kappa B (NF-kB)

The activation of NF-kB was measured using the commercially available chemiluminescent TransAM™ NF-kB transcription factor kit that measures the NF-kB p65 subunit (Active Motif, Carlsbad, CA). The kit contains a 96-well plate coated with oligonucleotide containing a NF-kB consensus site (5'-GGGACTTTCC-3'). Only the active form of NF-kB (i.e. not bound to inhibitor iNF-kB) specifically binds to this oligonucleotide. Therefore, nuclear purification is not necessary for this assay because inactivated cytoplasmic NF-kB cannot bind to the immobilized sequence. A primary antibody that recognizes the p65 subunit epitope is used subsequently to the incubation with cellular extract, which is obtained using the buffers included in the kit. A horse-radish-peroxidase-conjugated secondary antibody is used for the chemiluminescence assay. A standard curve was generated using dilutions of the NF-kB standard protein (Active Motif, Carlsbad, CA). Results were expressed in ng/μL.

### Statistical Analysis

Descriptive statistics were generated and distributions of data were analyzed using commercial software (PROC Univariate, SAS Institute, Version 9.1, Cary, NC). Box and Whiskers plots were produced using commercial software (KaleidaGraph, Version 4.01, Synergy Software, Reading, PA). Box plots represent the data collected. The box includes 50% of the observations with the top line indicating the upper quartile, the middle line showing the median value, and the lower line indicating the lower quartile. The lines extending from the box ("whiskers") mark the maximum and minimal values observed that are not outliers. Outliers are depicted by circles are a values that are either greater than the upper quartile + 1.5* the interquartile distance (ICD) or less than the lower quartile – 1.5*ICD. Non-normally distributed data was analyzed using non-parametric techniques (i.e. Kruskal-Wallis and Wilcoxin rank-sum, or Wilcoxin signed-rank depending on the number of comparisons and/or independence of observations) performed by commercially available software (PROC Npar1way, SAS Institute, Version 9.1, Cary, NC). General linear regression was used to examine the association between cell surface marker expression and age (PROC Reg, SAS Institute, Version 9.1, Cary, NC). The level of significance was set at p < 0.05.

## Results

### Effect of CpG-ODN 2135 in peripheral blood mononuclear cells of foals and adult horses

In a pilot study, we tested the proliferative response of 2-day-old foal (n = 5) and adult horse (n = 5) isolated peripheral blood mononuclear cells, and a 5-day-old foal isolated mesenteric lymph node mononuclear cells (n = 1) to CpG-ODN 2135 or non-stimulation. Those leukocytes included B cells and monocytes, which potentially express TLR9 and respond to CpG-ODN stimulation. Our results indicated that CpG-ODN 2135 motif induced proliferation of foal lymph node leukocytes *in vitro *with median stimulation indexes equal to 2 and 3 when cells were stimulated with 5 μg/ml or 10 μg/ml CpG-ODN 2135 final concentration, respectively, versus median stimulation index 0.8 when cells were stimulated with 12.5 μg/ml LPS. In addition, foal peripheral blood mononuclear cells responded to 10 μg/ml CpG-ODN or 12.5 μg/ml LPS with cell proliferation median stimulation indexes equal to 1.2 and 2.5, respectively. Adult horse cells presented median stimulation indexes 7.3 and 16.3, respectively.

### Cell culture system

**Figure 1:**
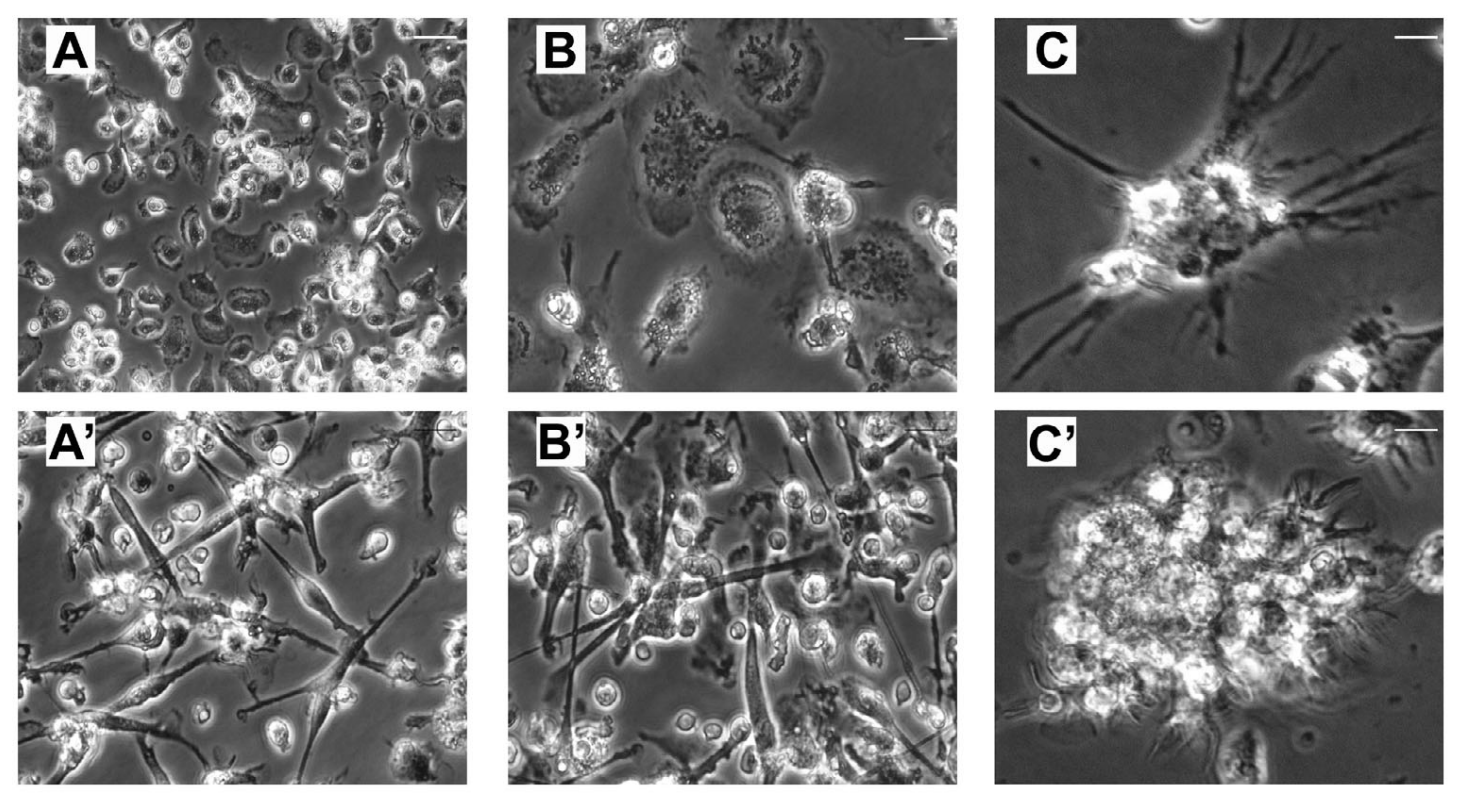
**Equine monocyte-derived macrophages (A) and dendritic cells (B) generated *ex vivo***. Isolated peripheral blood monocytes were stimulated (dendritic cells) or not (macrophages) with rEq IL-4 and rHuGM-CSF in DMEM-F12, 5% bovine growth serum. The photomicrogaphs depict the differentiation of adult horse and foal macrophages and dendritic cells in culture. A and B = day 5 adult horse and foal macrophages, respectively; A' and B' = day 5 adult horse and foal dendritic cells, respectively – note their extended shape in contrast to the round macrophages; C = day 6 dendritic cells adhered to the plastic of the cell culture plate; C' = a group of day 6 dendritic cells floating in the supernatant of the cell culture – note the presence of small dendrites. Bars indicate 50 μm.

Our *ex vivo *propagated adult horse monocyte-derived macrophages and DCs on Day 5 of culture exhibited a similar surface antigen phenotype to the one described by Hammond et al. [26] and Mauel et al. [33]. On day 5 of culture, adult horse and foal macrophages appeared round and attached to the plastic bottom of the culture plate (Figure 1). Foal macrophages tended to become giant cells more frequently in 2–3 month-old foal samples. In contrast, the adult horse and foal dendritic cells were elongated. After stimulation (day 6), occasional dendritic cells with stellate shape were observed, whereas many cells detached from the plastic, isolated or forming clumps, but keeping the dendrites.

**Figure 2:**
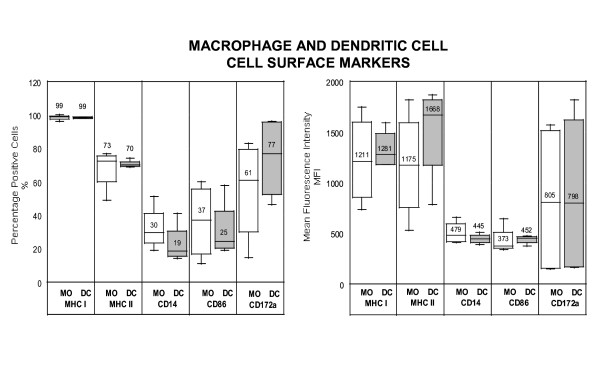
Percentage positive cells (%) and mean fluorescence intensity (MFI) of cell surface molecule expression in monocyte-derived macrophages (MO) and dendritic cells (DC) cultured for 5 days *ex vivo*. Note that immature dendritic cells revealed greater molecular expression (fluorescence intensity) for MHC class II and CD86 than macrophages, and inferior percentage of CD14-positive cells.

Approximately 30% and 19% of the monocyte-derived macrophages and DCs, respectively, expressed the CD14 marker. Approximately 61% and 77% of the monocyte-derived macrophages and DCs, respectively, expressed the CD172a marker. Overall, non-stimulated dendritic cells expressed 1.4 and 1.2 times median fluorescence intensity (hence molecular expression) for MHC class II and CD86, respectively, than macrophages (Figure 2). The percentages of CD8+ or CD4+ in rEqIL-4+rHuGM-CSF-stimulated cells were less than 3% and 9%, respectively. Foal cells presented similar phenotype to adult horse cells.

### Cell surface marker expression in stimulated and non-stimulated cells

**Figure 3:**
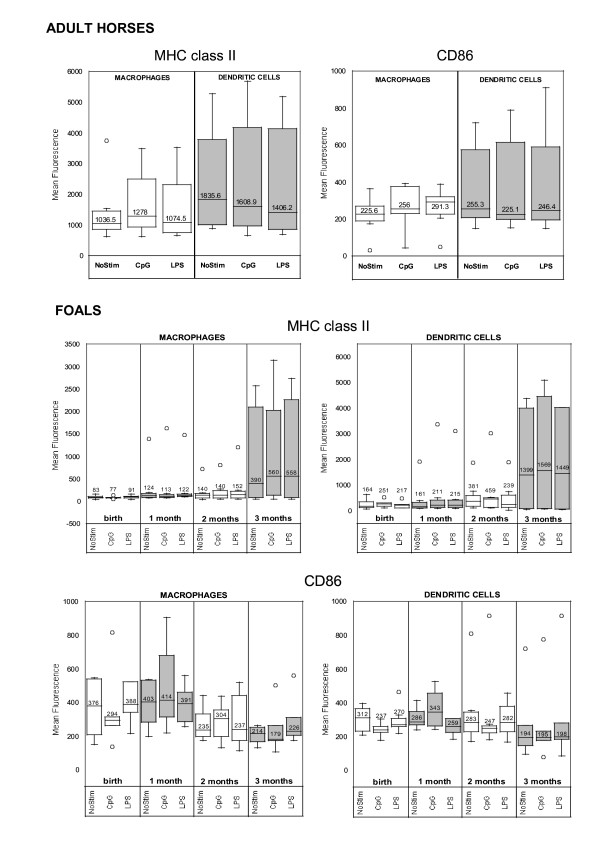
Mean fluorescence intensity (MFI) of cell surface molecule expression in monocyte-derived macrophages and dendritic cells stimulated with CpG-ODN for 14–16 hours after 5 days of culture *ex vivo*. Results are depicted for adult horses (A, n = 7) and foals (B, n = 7) of different ages. Although there was no specific effect of CpG-ODN or LPS stimulation in adult horse or foal cells, there was an age-dependent limitation in the expression of MHC class II on macrophage and dendritic cells of foals. The median fluorescences of the MHC class II molecule in non-stimulated foal macrophages and DCs at birth were 12.5× (p = 0.009) and 11.2× (p = 0.009) inferior, respectively, than adult horse cells, and 2.6× (p = 0.31) and 1.3× (p = 0.37), respectively, at 3 months of life.

Median fluorescence intensity of MHC class II expression was greater but not statistically significant different (p > 0.05) in DCs than in macrophages of adult horses and foals (Figure 3). Although there was no specific effect of CpG-ODN stimulation in adult horse and foal cells, there was an age-dependent limitation in the expression of MHC class II (fluorescence) on both macrophage and DCs of foals (p < 0.035). The median fluorescence of the MHC class II molecule in non-stimulated foal macrophages and DCs at birth were 12.5 times (p = 0.009) and 11.2 times (p = 0.009) inferior, respectively, to adult horse cells. At 3 months of life, there were no statistically significant differences in the expression of MHC class II molecule between foal and adult horse macrophages (2.6 times, p = 0.31) and dendritic cells (1.3 times, p = 0.37). The percentage of MHC class II positive cells remained somewhat constant through age. CpG-ODN or LPS treatment did not promote specific changes in MHC class II expression in macrophages or DCs, yet a statistically significant difference in MHC class II expression was observed in stimulated cells in an age-dependent in manner. The expression of the CD86 co-stimulatory molecule was comparable in adult horse and foal macrophages and DCs, independent of treatment.

### Cytokine mRNA expression in stimulated and non-stimulated cells

**Figure 4:**
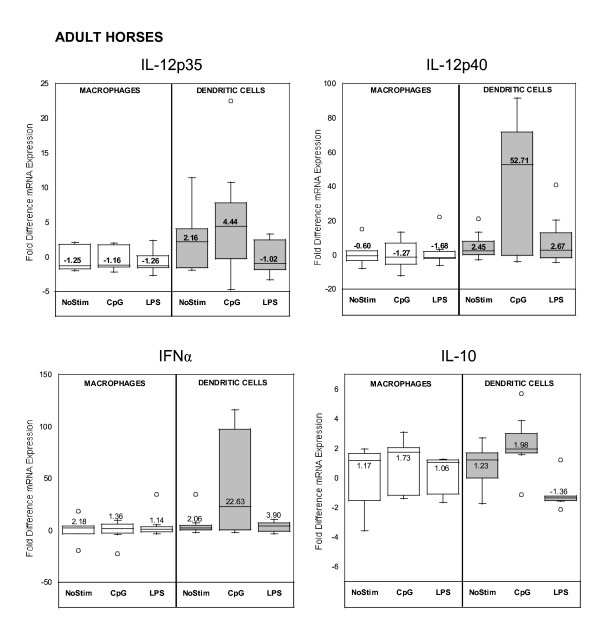
Quantitative cytokine (IL-12p35, IL-12p40, IFNα, IL-10) mRNA expression in adult horse (n = 7) monocyte-derived macrophages and dendritic cells stimulated or not (NoStim) with CpG-ODN or LPS for 14–16 hours after 5 days of culture ex vivo. Fold difference was calculated using baseline control values (non-stimulated cells on Day 5).

**Figure 5:**
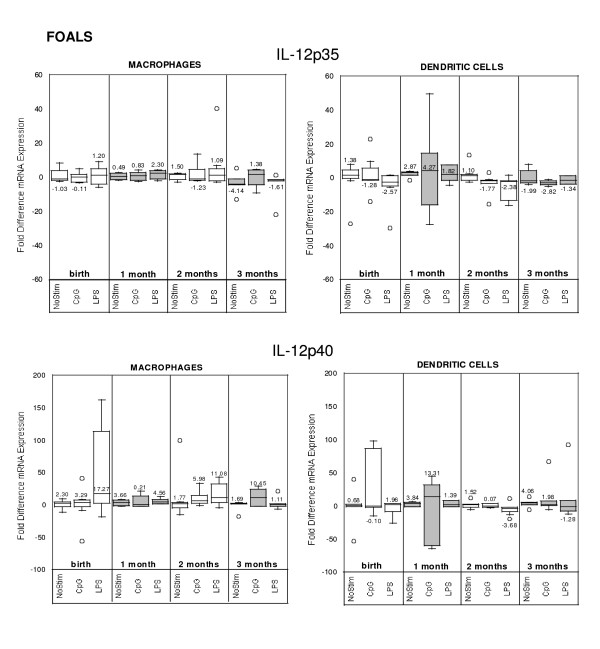
Quantitative cytokine (IL-12p35 and IL-12p40) mRNA expression in foal (n = 7; A = birth, B = 1 month, C = 2 months, D = 3 months) monocyte-derived macrophages and dendritic cells stimulated or not (NoStim) with CpG-ODN or LPS for 14–16 hours after 5 days of culture ex vivo. Fold difference was calculated using baseline control values (non-stimulated cells on Day 5).

**Figure 6:**
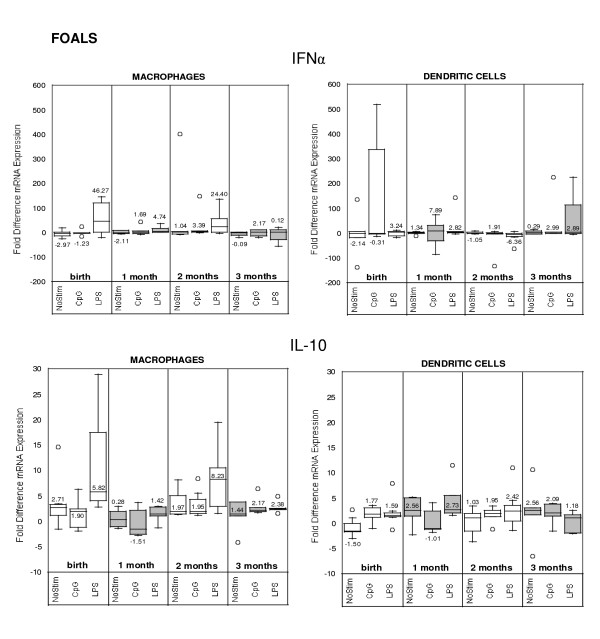
Quantitative cytokine (IFNα and IL-10) mRNA expression in foal (n = 7; A = birth, B = 1 month, C = 2 months, D = 3 months) monocyte-derived macrophages and dendritic cells stimulated or not (NoStim) with CpG-ODN or LPS for 14–16 hours after 5 days of culture ex vivo. Fold difference was calculated using baseline control values (non-stimulated cells on Day 5).

Adult horse DCs increased the median IL-12p40 and IFNα mRNA expression 53 and 23 times, respectively, upon CpG-ODN stimulation, in comparison to non-stimulated DCs (p = 0.078). Adult horse CpG-ODN-stimulated macrophages did not change their cytokine mRNA expression in comparison to non-stimulated cells (Figure 4). Foal APCs did not change mRNA cytokine expression in an age-dependent manner upon CpG-ODN stimulation up to 3 months of age; instead, random fold differences were observed in the data with both CpG-ODN and LPS stimulation (Figures 5 and 6). The expression of IL-12p40 and IFNα in adult horse non-stimulated DCs were comparable to foal DCs at birth (p > 0.05). Despite the distinct median values, there was not a statistically significant difference in CpG-ODN stimulated cells between both groups. In order to evaluate if LPS was inducing a different pattern of cytokine expression than CpG-ODN, we tested TNFα mRNA expression in a small subset of adult horse samples: at 14–16 hours, CpG-ODN-stimulated DCs revealed a 5-fold increase in comparison to non-stimulated DCs, whereas LPS-stimulated-DCs revealed a 1-fold decrease. Stimulated and non-stimulated macrophages did not show any differences in their TNFα mRNA expression.

### TLR9 and NF-kB signaling pathway

**Figure 7:**
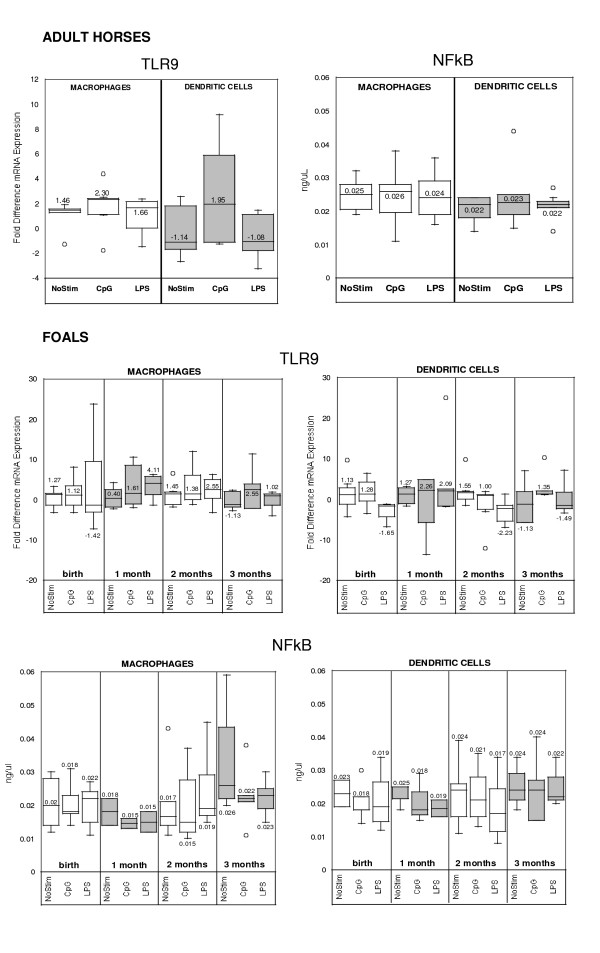
Quantitative analysis of TLR9 and NFkB p65 in monocyte-derived macrophages and dendritic cells stimulated with CpG-ODN or LPS for 14–16 hours after 5 days of culture ex vivo. Results are depicted for (A) adult horses (n = 7) and (B) foals of different ages (n = 7; A = birth, B = 1 month, C = 2 months, D = 3 months).

TLR-9 mRNA expression in foal DCs and macrophages were comparable (p > 0.05) to adult horse cells, and CpG-ODN treatment induced upregulation of a 1-fold difference in comparison to non-stimulated and LPS-stimulated cells (Figure 7). Values for NF-kB activation (NF-kB p65) were comparable (p < 0.05) in adult horse and foal macrophages and DCs, independent of treatment.

## Discussion

### Age-dependent aspects of APCs in the horse

Limitations in the immune system of the foal could be associated with age-dependent development of cell interaction for a primary immune response. The low expression of MHC class II in equine neonate and young foal peripheral blood lymphocytes has been well documented, but the expression of this essential molecule in APCs had not been studied before in the foal [34,35]. Our investigation revealed 2 important observations: a) there was a statistically significant difference in the fluorescence expression of MHC class II in macrophages and DCs of foals with age; and b) median MHC class II fluorescence expression in non-stimulated macrophages and DCs of the foal at birth were 12.5 times and 11.2 times inferior, respectively, to adult horse cells. The median MHC class II fluorescence expression in non-stimulated DCs of 3 month-old-foals was comparable to adult horses, which suggests a greater competence for the priming of T cells at that age. In human fetuses, the percentage of MHC class II-positive monocytes increases significantly over gestation but remains lower than the adult human at term [36]. Limitation in APC number and function in young age has been shown to contribute to poor protective cellular immune responses [37-39]. Human cord blood DCs are less efficient in the activation of T cells *in vitro *and instruction to a Type 1 immune response, likely due to their lower cell surface MHC class I and II, co-stimulatory (CD86), and adhesion molecule expression levels than adult human blood cells [40].

Likewise, the expression of cytokines and co-stimulatory molecules (signal II) in APCs had not been studied before in foals. These important immune mediators are critical for the priming and clone expansion of naïve T cells. There were no statistically significant differences in the expression of CD86 in foal macrophages and DCs. In addition, there were no age-dependent changes in the expression of CD86. Importantly, those values were comparable to the adult horse, and they suggest that APCs of foals are competent in the expression of the CD86 co-stimulatory molecule.

### Response to stimulus

CpG-ODN 2135 was a functional tool to evaluate the innate immune response in foals, and to compare those results to adult horse response. We learned that adult horse DCs, but not macrophages, increased the IL-12p40 and IFNα mRNA expression 53 and 23 times, respectively, in comparison to non-stimulated DCs, whereas foal DCs did not respond specifically to that stimulus up to 3 months of life. Despite the lack of statistical difference, the contrast between foal and adult horse cell cytokine responses to CpG-ODN should not be overlooked, but further pursued for better understanding of foal response to different types of pathogens and vaccines/adjuvants. Other CpG-ODN motifs could induce different types and magnitude of response by adult horse and foal cells. However, the CpG-ODN motif used herein revealed a difference between adult horse and foal DC response. Indeed, in our pilot studies, this same CpG-ODN induced greater proliferation indexes in adult horse peripheral blood leukocytes than foal cells.

Interleukin-12 is a heterodimeric molecule composed of p35 and p40 subunits. Upon CpG-ODN stimulation, adult horse DCs increased the expression of IL-12p40, which was not matched in magnitude by IL-12p35. Holscher et al. [41] demonstrated a protective and agonistic role of IL-12p40 in mycobacterial infection in IL-12p35 knockout mouse. This immune effect could have been associated with the expression of IL-23, which comprises the same p40 subunit of IL-12 but a different p19 subunit. Therefore, it is possible that the IL-12p40 response to CpG-ODN in adult horse DCs may reflect the expression of IL-23, instead, and that needs to be tested. Whereas IL-12 promotes the development of naïve T cells, IL-23 participates in the activation of memory T cells and chronic inflammation, and this difference is relevant when studying the development of primary immune response in foals [42].

Both IL-12 and IFNα promote activation of T cells into Type 1 immune response, with activation, proliferation and IFNγ production [43,44]. Subsequently, CD40-ligand engagement and IFNγ from activated T cells facilitate the production of IL-12 by APCs [45,46]. Indeed, mouse conventional DCs require IFNγ co-stimulation for the production of the active form of IL-12 upon TLR stimulation [47]. Therefore, an impaired cytokine signaling for appropriate APC activation in foals could not only hamper a subsequent Type 1 primary immune response, but also the proper activation of APCs. In fact, this may be a limiting factor in foals because Breathnach et al. [48] have demonstrated that the equine neonate peripheral blood and pulmonary lymphocytes present a marked low response for the production of IFNγ, which improves steadily with age.

Compromised Th1 differentiation has been also observed when there is CD4+ T cell hyporesponsiveness to IL-12 [49]. In young age, DC maturation and cytokine production may require specific and co-stimulatory stimuli, which may become less crucial in a more developed (adult) immune system. In addition, IL-12 production can be antagonized by the presence of the anti-inflammatory cytokines IL-10 and TGFβ [50]. Foal DCs did not alter the expression of IL-12 upon stimulation; yet, those cells did not change the expression of IL-10 either. Therefore, it is unlikely the lack of IL-12 response was due to a bias of the foal cells toward an anti-inflammatory state; rather, it is possible that those cells have a decreased overall response to stimulus up to 3 months of life through the TLR9 signaling pathway [51].

Similarly to CpG-ODN, LPS has been shown to induce DC maturation with cytokine production, up-regulation of co-stimulatory molecules and activation of T cells. Those effects were not observed in our data. LPS inflammatory stimulation involves both common and different pathways to CpG-ODN, and distinct cytokine expression kinetics has been observed [17,52]. To investigate whether LPS was inducing a different pattern of cytokine response, we evaluated the TNFα mRNA expression in a subset of adult horse samples. At 14–16 hours of stimulation, CpG-ODN- or LPS-stimulated DCs expressed TNFα mRNA with a median 5-fold increase and 1-fold decrease, respectively, in comparison to non-stimulated cells. It is possible that the peaks of cytokine expression of LPS-stimulated DCs were missed by the time the cells were harvested, and measuring protein levels would have been a better comparison.

Two classes of CpG have been described to induce different effects in human cells: CpG-A and CpG-B. The former has a phosphodiester core with CpG motifs, flanked by phosphorothioate poly(G) sequences on both the 3' and 5' ends; the latter is mainly a phosphorothioate, nuclease resistant backbone [53,54]. CpG-A had been originally known to stimulate plasmacytoid DCs to express large amounts of IFNα; and CpG-B as a potent stimulator of B cell proliferation and secretion of IL-10 [1,3,55,56]. Both types of CpG require TLR9 for immune stimulation [57]. However, only CpG-B has been shown to activate NF-kB, whereas CpG-A induces a minimal response [58]. In our studies, median TLR9 expression was comparable in CpG-ODN-treated or LPS-treated macrophages and DCs of foal and adult horse cells. NF-kB activation in foal macrophages and DCs was comparable to adult horse cells, and CpG-ODN or LPS treatment did not reveal an effect in any of the groups. Therefore, those analyses were not informative of the mechanisms involved in cell activation upon CpG-ODN stimulation.

Structurally, the CpG-ODN used in these experiments is of class B. However, its effect on horse cells resembled the one of class A in other species for the increased IFNα expression and lack of concomitant increased expression of NF-kB in the adult horse dendritic cells. Distinct responses to CpG-ODN have been described in different species. Mena et al. [59] have shown a specific and dose-dependent IFNα response to class B CpG-ODN motif-stimulated ovine, but not bovine, peripheral blood mononuclear cells. In addition, class B CpG-ODN has been shown to induce *in vitro *IFNα production in newborn lambs, which seems to contrast with our findings in foals [60]. Nevertheless, it is possible that IFNα expression in equine cells is higher when cells are stimulated with class A CpG-ODN. Wattrang et al. [19] demonstrated that class A CpG-ODN indeed induces IFNα expression by equine peripheral blood mononuclear cells.

The maturation of DCs measured by MHC class II expression upon CpG-ODN stimulus was not obvious in adult horse cells, potentially because those cells were already expressing high levels of that molecule on the cell surface on Day 5 of the *ex vivo *culture. Alternatively, there were mixed-maturation stage cells in the cell culture well, and only a fraction of those cells became mature with greater MHC class II expression. Our flow cytometric analysis for MHC class II expression did not include specific gated areas in the DC population to keep consistent with the mRNA cytokine data, which was generated from the whole cell population. Yet, a subpopulation of cells with high side and forward scatters in the dot plots expressed the highest levels of MHC class II, and CpG-ODN stimulation could have induced distinct increased expression of that molecule in comparison to controls.

### Categorization of the monocyte-derived macrophages and dendritic cells

The *ex vivo *model presented here produced monocyte-derived macrophages and DCs with characteristics comparable to published results [26,33,61,62]. On Day 5 of cell culture, rEqIL-4 + rHuGM-CSF induced a slight increase in the expression of MHC class II molecule (fluorescence), whereas the number of cells (percentage) expressing CD14 molecule was decreased in comparison to control. Those results suggest the generation of immature DCs, which were desired for our experiments. Nevertheless, it is unlikely that this system produced macrophage or DC cell populations in synchronous stages of development. Both macrophages and DCs were derived primarily from adherent peripheral blood mononuclear cells, and a high percentage of cells expressing the CD172a molecule was present in the cell culture. Although CpG-ODN may not have induced DC maturation per se as it is classically measured (i.e. increased MHC class II expression), only stimulated DCs (and not non-stimulated DCs and stimulated macrophages) induced IL-12p40 and IFNα cytokine expression.

The classification of DCs is quite complex: the heterogeneity of DCs is determined by the precursor population, anatomical localization, function, and the final outcome of the immune response [15,63]. Several DC subsets have been identified in human and mouse, and some similarities and differences exist between species [64]. Two major categories, conventional DCs or plasmacytoid DCs, can be described according to the cell origin, TLR expression and cytokine profile. The cell surface marker CD11c has been an important parameter in the identification of DCs; however a monoclonal antibody that recognizes this marker is lacking for the equine species. In general, conventional DCs express TLR4 and plasmacytoid DCs express TLR9, and other TLRs may or not be expressed in the same cell types in both species [65]. In addition, conventional DCs are known to produce high levels of IL-12, whereas plasmacytoid DCs produce type I IFN (IFNα) and IL-12 [16].

To date, there is no single reliable method for the characterization and categorization of equine DCs derived from peripheral blood or from peripheral or lymphoid tissues. Therefore, the combination of cell surface marker expression, using the monoclonal antibodies available for the horse species, and the expression of cytokines upon stimulation may reveal preliminary characteristics of those cells. It is not clear from our analyses if the cells producing IFNα and IL-12 were positive or not for the CD172a and CD14 markers. This question would require a double staining of cytokines and cell surface markers, and those reagents are not widely available for horse proteins to this date. Alternatively, this system generates a type of DC that does not follow a predetermined classification system, such as the one described by Asselin-Paturel et al. [66], a unique subset of murine immature APCs with plasmacytoid morphology that secrete IFNα and IL-12 upon stimulation with viruses and CpG-ODN.

## Conclusion

The results from our *ex vivo *system suggest that foal APCs do not respond to stimulus comparably to adult horse cells in cytokine expression. In addition, this investigation revealed an age-dependent limitation in the expression of MHC class II molecule in the APCs of the newborn and young foal, although the expression of the co-stimulatory molecule CD86 seems to be present already in early life. Our studies are not comprehensive in determining the intrinsic developmental aspects of the foal APCs, yet they bring new observations to support future studies in the competence of the foal cells to elicit a primary immune response, and in the choice of appropriate adjuvants for use in young age. CpG-ODN has shown positive effects in DC maturation and activation in neonatal cells of other species. In addition, different CpG-ODN motifs have distinct effect in immune cells. Other types of stimulants (e.g. inactivated whole Gram positive or negative organisms, inactivated viruses, or distinct CpG-ODN motifs) may further indicate levels of response, and potential limitations of APCs to signal T cells for a primary immune response in young age.

## Competing interests

The author(s) declare that they have no competing interests.

## Authors' contributions

MJBFF conceived the study design, coordinated the study, performed the blood collection, and flow cytometric analysis. MBM performed the cell culture, cell harvesting and freezing. ASB performed the RNA isolation, real-time quantitative RT-PCR, and chemiluminescence assay. DWH provided technical orientation and reagents for the cell culture. RH determined and provided the motif to be used in the experiments. MJBFF and ASB prepared the draft of the manuscript. DVN and ASB performed the data analysis. All authors read and contributed to the final version of the manuscript.
